# Inhibition of *Cronobacter sakazakii* by *Litsea cubeba* Essential Oil and the Antibacterial Mechanism

**DOI:** 10.3390/foods11233900

**Published:** 2022-12-02

**Authors:** Haoran Wang, Yulu Li, Zhuo Li, Run Ma, Xiangyang Bai, Xiangjun Zhan, Kunyao Luo, Ruiying Su, Xuejiao Li, Xiaodong Xia, Chao Shi

**Affiliations:** 1 College of Food Science and Engineering, Northwest A&F University, Xianyang 712100, China; 2 National Engineering Research Center of Seafood, School of Food Science and Technology, Dalian Polytechnic University, Dalian 116304, China

**Keywords:** antimicrobial activity, *Litsea cubeba* essential oil, *Cronobacter sakazakii*, biofilm, confocal laser scanning microscopy

## Abstract

*Litsea cubeba* essential oil (LC-EO) has anti-insecticidal, antioxidant, and anticancer proper-ties; however, its antimicrobial activity toward *Cronobacter sakazakii* has not yet been researched extensively. The objective of this study was to investigate the antimicrobial and antibiofilm effects of LC-EO toward *C. sakazakii*, along with the underlying mechanisms. The minimum inhibitory concentrations of LC-EO toward eight different *C. sakazakii* strains ranged from 1.5 to 4.0 μL/mL, and LC-EO exposure showed a longer lag phase and lower specific growth compared to untreated bacteria. LC-EO increased reactive oxygen species production, decreased the integrity of the cell membrane, caused cell membrane depolarization, and decreased the ATP concentration in the cell, showing that LC-EO caused cellular damage associated with membrane permeability. LC-EO induced morphological changes in the cells. LC-EO inhibited *C. sakazakii* in reconstituted infant milk formula at 50 °C, and showed effective inactivation of *C. sakazakii* biofilms on stainless steel surfaces. Confocal laser scanning and attenuated total reflection–Fourier-transform infrared spectrometry indicated that the biofilms were disrupted by LC-EO. These findings suggest a potential for applying LC-EO in the prevention and control of *C. sakazakii* in the dairy industry as a natural antimicrobial and antibiofilm agent.

## 1. Introduction

Among the *Enterobacteriaceae*, *Cronobacter sakazakii* is a ubiquitous opportunistic foodborne pathogen that is Gram-negative, facultatively anaerobic, and nonspore-forming [1]. *Cronobacter sakazakii* is found in foods such as milk, cheese, vegetables, meat, bread, and tea [2]. Existing studies have shown that the contamination rate of *C. sakazakii* to PIF varied between 2% and 14% [3]. The overall incidence of *C. sakazakii* infections was reported to be 0.66 cases/100,000 population, which was higher than anticipated [4]. It is associated with severe infections in newborns and infants; neonatal meningitis, septicemia, and necrotizing enterocolitis can be caused by *C. sakazakii*, with an 80% mortality rate for neonatal infections [5]. Epidemiological studies have shown that *C. sakazakii* infection in infants is closely associated with contaminated powdered infant formula (PIF) [1]. The bacteria can survive in PIF for >2 years, posing a great risk to the health of newborns [6].

*Cronobacter sakazakii* can also adhere to solid surfaces and form biofilms, which increases its resistance to sterilization measures [7]. According to Beuchat et al. [8], *C. sakazakii* cells in infant formula broth possessed the ability to adhere to the container and form biofilms. *Cronobacter sakazakii* can also form biofilms on the esophagus of newborns, which may cause neonatal infection [9]. Moreover, *C. sakazakii* has the ability to attach to and form biofilms on multiple abiotic materials, including latex, polycarbonate, silicon, glass, stainless steel, and other food container materials [8].

During the processes of PIF production, drying, filling, and packing are likely to come into contact with bacteria in the air and packaging materials, which can lead to *C. sakazakii* contamination in the environment [10]. During storage, PIF is at increased risk of *C. sakazakii* contamination because of factors including exposure to variable temperatures, humidity, and storage conditions [11]. Traditional thermal treatment, including melting the PIF with boiling water, have been used to reduce the occurrence of *C. sakazakii* in reconstituted infant formula (RIF); however, high temperatures can cause the destruction of nutrients [12]. For biofilms, chemical treatment with agents, such as hydrogen peroxide and sodium hypochlorite, is a conventional sterilization method to control biofilms of some foodborne pathogenic bacteria [13]. However, there is a growing concern among consumers regarding the safety of chemical agents [14]. Natural methods are a viable alternative to reduce or eliminate the negative effects of chemical treatments in the production process of food [15]. It is necessary to develop a highly effective natural bacteriostatic agent against *C. sakazakii* contamination. In recent years, natural plant essential oils have been investigated as antibacterial agents [16].

Essential oils, including *Litsea cubeba* essential oil (LC-EO), are generally recognized as safe (GRAS), and are used as natural antibacterial agents [17]. LC-EO is mainly extracted from *Litsea cubeba* fruit, and its application prospects in the food, cosmetics, and pharmaceutical industries are wide [18]. Recent studies have described the health-related functions of LC-EO added to food, such as anti-insecticidal, antioxidant, and anticancer properties [19]. Additionally, LC-EO has antiviral, antifungal, and antibacterial effects [18]. In terms of bacteria, LC-EO has antibacterial effects on Gram-positive bacteria, such as *Salmonella* [20] and *Escherichia coli* O157:H7 [18], and Gram-negative bacteria, such as methicillin-resistant *Staphylococcus aureus* [21], have been reported. However, its antibacterial effects toward *C. sakazakii*, especially in RIF, have yet to be tested., and there are few reports of its effects on bacterial biofilms [20].

The objectives of this study were as follows: (i) to evaluate the antibacterial activity and the possible mechanisms of action of LC-EO toward *C. sakazakii*; (ii) to measure the efficacy of LC-EO combined with mild heating against *C. sakazakii* in RIF; and (iii) to assess the ability of LC-EO to eliminate *C. sakazakii* biofilms on stainless steel surfaces.

## 2. Materials and Methods

### 2.1. Chemicals, Bacterial Strains, and Growth Conditions

LC-EO (high-performance liquid chromatography ≥95%, CAS 68855-99-2) was acquired from Shanghaiyuanye Bio-technology Co., Ltd. (Shanghai, China). Dimethyl sulfoxide (DMSO) was used to dissolve LC-EO. The final concentration of DMSO in sample solutions was 0.5% (*v*/*v*). Land Bridge Technology Co., Ltd. (Beijing, China) provided tryptic soy broth (TSB) and tryptic soy agar (TSA). The composition of TSB is 1.5% of tryptone, 0.5% of soybean peptone, 0.5% of sodium chloride, prepared with distilled water, adjusted pH 7.2 ± 0.2, and sterilized by 121 °C pressure steam. TSA is 1.5% agar added to TSB. All other chemicals were maintained in the analytical grade.

Eight strains of *C. sakazakii* were used in the present study. *Cronobacter sakazakii* ATCC 29004, ATCC 29544, ATCC 12868, and ATCC BAA-894 were obtained from the American Type Culture Collection (ATCC; Manassas, VA, USA). Four other strains of *C. sakazakii* were selected from our laboratory strain collection [7]: two (strains 14-15(1) and 18-8(2)) were originally isolated from RIF, and the other two (strains 11-8(2) and 18-7(2)) were originally isolated from rice flour. Minimum inhibitory concentration (MIC) and minimum bactericidal concentration (MBC) values for all strains were determined, and the following experiments were conducted using *C. sakazakii* ATCC 29004.

Experimental strains were precultured on TSA for 24 h at 37 °C in aerobic conditions as previously described [7]. All strains were inoculated into sterile TSB and incubated at 37 °C for 24 h with shaking at 130 rpm.

### 2.2. Determination of MIC and MBC

According to the broth microdilution method described in the Clinical and Laboratory Standards Institute guidelines [22], with slight modifications, the MIC values were determined. In brief, bacterial suspensions were diluted to 5 × 10^5^ CFU/mL in TSB. Then, in a 96-well microtiter plate, equivalent volumes (100 µL) of the bacterial suspension and serial dilutions of LC-EO in TSB were mixed into the wells to obtain final concentrations of 8, 6, 4, 3, 2, 1.5, and 0 (Control) µL/mL. A microtiter plate reader (model 680; Bio-Rad, Hercules, CA, USA) was used to determine the optical density in each well at 600 nm (OD_600 nm_). The measurement was then repeated after 24 h of incubation at 37 °C. The MIC of LC-EO was recorded as the minimum antimicrobial concentration that resulted in the change in OD_600 nm_ < 0.05.

MBC was identified following the MIC assay. Briefly, 100 μL aliquots from wells that did not grow were plated on TSA and incubated at 37 °C for 24 h, and the colonies were enumerated. The MBC was recorded as the minimum concentration with no colony growth.

### 2.3. Growth Curves

The growth curves of were determined as previously described [23] with minor modifications. *Cronobacter sakazakii* cultures were diluted to 5 × 10^5^ CFU/mL in TSB with (1/16, 1/8, 1/4, 1/2, and 1MIC) or without LC-EO and incubated at 37 °C, and the growth of *C. sakazakii* was monitored by measuring OD_600 nm_ every hour for 24 h using a microtiter plate reader (model 680).

### 2.4. Reactive Oxygen Species (ROS)

According to Li et al. [24], intracellular ROS levels were detected by the fluorescent molecule dichlorodihydrofluorescein diacetate (DCFH-DA; Beyotime Institute of Bio-technology, Shanghai, China). *Cronobacter sakazakii* ATCC 29004 suspension was diluted to 10^7^ CFU/mL in phosphate-buffered saline (PBS, 135 mmol/L NaCl, 2.7 mmol/L KCl, 1.5 mmol/L KH_2_PO_4_, and 8 mmol/L K_2_HPO_4_, pH 7.2). Then, the cells were incubated with the addition of DCFH-DA (5 μmol/L, final) for 10 min at 37 °C. Next, LC-EO was added (0, 1/8, 1/4, 1/2, and 1MIC, final) and the mixture was incubated at 37 °C for 10 min. After incubation and washing with PBS, the fluorescence intensity (excitation 488 nm, emission 525 nm) was detected by a fluorescence microplate reader (SpectraMax M2, Molecular Devices, San Jose, CA, USA), and the data were normalized by cell numbers.

### 2.5. Membrane Integrity

*Cronobacter sakazakii* ATCC 29004 suspensions (~10^8^ CFU/mL) were treated with LC-EO (0, 1/4, 1/2, and 1MIC, respectively) at 37 °C for 30 min. After treatment, these samples were centrifuged (10,000× *g*, 2 min, 4 °C), and the cell pellets were resuspended in 2 mL of 0.85% (*w*/*v*) NaCl solution. A live/dead bacterial double stain kit (Shanghai Maokang Biotechnology Co., Ltd., Shanghai, China) was used, and, in accordance with the manufacturer’s instructions, 1.5 μL of fluorescent dyes SYTO 9 and 1.5 μL of propidium iodide (PI) were prepared and mixed. Then, 3 µL of the mixed reagent was added to a 1 mL aliquot of each cell suspension, and cultured in the dark for 10 min at room temperature. The samples were observed via confocal laser scanning microscopy (CLSM; A1, Nikon, Tokyo, Japan) under 300× magnification.

### 2.6. Membrane Potential

The membrane potential of *C. sakazakii* ATCC 29004 was determined according to the method described by Shi et al. [23]. Cells were diluted to 1 × 10^8^ CFU/mL in PBS. Then, 125 μL cell suspensions were added into black 96-well microtiter plates (Nunc, Copenhagen, Denmark). Every cell suspension well was treated with the membrane-potential-sensitive fluorescent probe *bis*-(1,3-dibutyl barbituric acid) trimethine oxonol (DiBAC_4_(3); Molecular Probes, Eugene, OR, USA) for 30 min at 37 °C in the dark. The final concentration of DiBAC_4_(3) was 1 μmol/L. Then, 125 μL of LC-EO was added to wells to final concentrations of 0, 1, and 2MIC. With reference to the parameter setting of Shi et al. [23], the fluorescence of *C. sakazakii* was recorded by a fluorescence microplate reader (excitation 492 nm, emission 515 nm; SpectraMax M2, Molecular Devices, San Jose, CA, USA).

### 2.7. Intracellular ATP

The intracellular ATP of *C. sakazakii* ATCC 29004 was determined as described by Shi et al. [25], with some modifications. Briefly, *C. sakazakii* were resuspended in PBS at approximately 10^8^ CFU/mL, the bacterial suspensions were treated with LC-EO at different concentrations (0, 1, and 2MIC) at 37 °C for 30 min. Then, each sample was ultrasonicated on ice, centrifuged (5000× *g*, 4 °C, 5 min), and the supernatant was retained. An ATP assay kit (Beyotime Bioengineering Institute, Shanghai, China) was used to measure ATP concentration. The supernatant (100 μL) was mixed with prepared ATP detection reagent in a white 96-well microtiter plates, and the bioluminescence of each well was determined by a multimode microplate reader (Spectra Max M2). ATP standard solutions with known concentrations (0.01, 0.1, 1, and 10 μmol/L) were used to obtain a standard curve, and the intracellular ATP concentration was calculated according to the standard curve.

### 2.8. Bacterial Morphology

Field-emission scanning electron microscopy (FESEM) was applied according to a previously published method of Subramaniyan et al. [26], with slight modifications. Cultures of *C. sakazakii* ATCC 29004 with and without LC-EO treatment (1/4, 1/2, and 1MIC) were incubated at 37 °C for 30 and 60 min. Then, they were washed with PBS and fixed for 12 h at 4 °C with 2.5% (*v*/*v*) glutaraldehyde. The bacteria were dehydrated step-by-step with alcohol solutions (30%, 50%, 70%, 80%, 90%, 100%, 10 min each) with shaking. Finally, after air drying, the samples were sputtered with gold and observed under a FESEM at 15,000× magnification (S-4800, Hitachi, Tokyo, Japan).

### 2.9. Antibacterial Activity of LC-EO in TSB and PBS

LC-EO was added to suspensions of *C. sakazakii* ATCC 29004 (6.5 log CFU/mL) in TSB or PBS, and the final concentrations of LC-EO were 0 (Control), 1/4, 1/2, and 1MIC. The samples were incubated for 0, 0.5, 1, 2, 4, 6, and 8 h. Then, decimal dilutions of the suspension were incubated on TSA; plates were incubated at 37 °C for 24 h prior to enumeration.

### 2.10. Antibacterial Activity of Combined Treatment with LC-EO and Mild Heat in RIF

PIF was purchased from a local market (Yangling, Shaanxi, China). The background bacterial cells in PIF were completely killed by irradiation at 4 kGy for 2 s. RIF was reconstituted according to the instructions on the label, with 13.5 g powder was rehydrated in 90 mL sterile distilled water (SDW). Then, *C. sakazakii* suspensions (ATCC 29004, 14-15(1), and 18-8(2)) were added to the RIF to approximately 6.5 log CFU/mL. LC-EO was added to the samples, and the final concentrations of LC-EO were 0 (Control), 5, 10, and 20MIC. The samples were incubated at 25 or 50 °C. Aliquots were taken from each sample at 0, 20, 40, 60, 80, 100, and 120 min and plated on TSA. The plates were then incubated for 24 h at 37 °C prior to enumeration.

### 2.11. Inactivation Effect of LC-EO on C. sakazakii in Biofilms

*Cronobacter sakazakii* ATCC 29004 culture was diluted in TSB to 10^7^ log CFU/mL. In the 24-well plates containing sterile stainless steel coupons (type 304; finish 4; 1 cm × 1 cm × 0.1 cm), 2 mL aliquots of *C. sakazakii* ATCC 29004 were added and placed at 25 °C for 48 h for biofilm formation. Subsequently, the steel coupons were washed with SDW and treated with LC-EO at 0, 5, 10, and 20MIC at 25 °C. The prepared samples were incubated for 0, 0.5, 1, 1.5, 2, 2.5, 3, 3.5, and 4 h. Then, after being washed with SDW, the coupons were placed in 4 mL centrifuge tubes containing 2 mL of PBS and 0.5 g of glass beads (G8772, 425–600 μm; Sigma-Aldrich, St. Louis, MO, USA) and vortexed for 1 min. Finally, to measure the concentration of the viable cells, the samples were incubated on TSA for 24 h at 37 °C.

### 2.12. Effect of LC-EO on Matrix Components in C. sakazakii Biofilms

Biofilms were formed as described in Section 2.11. The biofilm was aseptically washed three times with SDW to remove unattached cells, treated with LC-EO (5, 10, and 20MIC for 1 h), and then washed with SDW. Biofilms were scanned in an attenuated total reflection–Fourier-transform infrared (ATR-FTIR) spectrometer (NEXUS 670, Thermo Nicolet, Waltham, MA, USA) from 1700 to 800 cm^−1^ with 16 scans and 4 cm^−1^ resolution. In accordance with Wang et al. [27], peaks were assigned to proteins and polysaccharides.

### 2.13. CLSM-Based Analysis of the Antibiofilm Activity of LC-EO

CLSM was used to observe the mature biofilms established as described in Section 2.11 after treatment by LC-EO at 0, 10, and 20MIC at 25 °C for 10 min. As described by Yang et al. [28], after washing with SDW, the biofilms were dyed with concanavalin A-fluorescein isothiocyanate (Con A-FITC; Shanghai Maokang Biotechnology Co., Ltd., Shanghai, China) for 15 min at 4 °C and immobilized by 2.5% (*v*/*v*) glutaraldehyde solution for 2 h at 4 °C. Then, the biofilms were dyed with Hoechst 33258 for 20 min. All operations were performed in the dark. The coupons were observed using CLSM (A1) at 40× magnification. Imaging was performed with 488 nm excitation of Con A-FITC and 405 nm excitation of Hoechst 33258, respectively.

### 2.14. Statistical Analysis

Each assay was independently performed in triplicate. Statistical differences were analyzed using IBM SPSS software version 16.0 (SPSS Inc., Chicago, IL, USA). Results were expressed as the mean ± standard deviation. Student’s *t*-test was used to test significant differences between control and treated samples. For all statistically significant analyses, *p* needed to be less than 0.05.

## 3. Results

### 3.1. MIC and MBC of C. sakazakii by LC-EO

Four ATCC standard strains and four strains from our laboratory collection were selected to determine the MIC and MBC of LC-EO toward *C. sakazakii* (Table 1). The MIC of LC-EO ranged from 3.0 to 4.0 μL/mL, except for isolate 14-15(1), for which the MIC was 1.5 μL/mL. The MBC of LC-EO ranged from 3.0 to 4.0 μL/mL, except for isolates 18-7(2) and 11-8(2), for which the MBC was 6.0 μL/mL. As a reference strain, *C. sakazakii* ATCC 29004 was selected for further experiments.

### 3.2. Effects of LC-EO on C. sakazakii Growth

**Figure 1:**
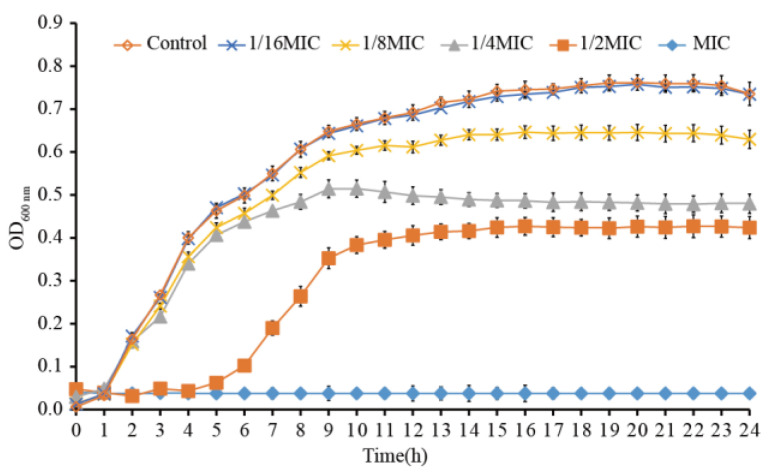
Growth curves for *C. sakazakii* in TSB with various concentrations of LC-EO.

When LC-EO concentration was equal to or higher than the MIC, the growth of *C. sakazakii* ATCC 29004 was completely inhibited (Figure 1). The lag period of *C. sakazakii* growth was prolonged when the LC-EO concentration was 1/2MIC, and the maximum bacterial concentration was reduced in an LC-EO-concentration-dependent way from 0MIC (Control) to 1/2MIC. The *C. sakazakii* growth was almost unaffected when the LC-EO concentration was 1/16MIC.

### 3.3. ROS Analysis

**Figure 2:**
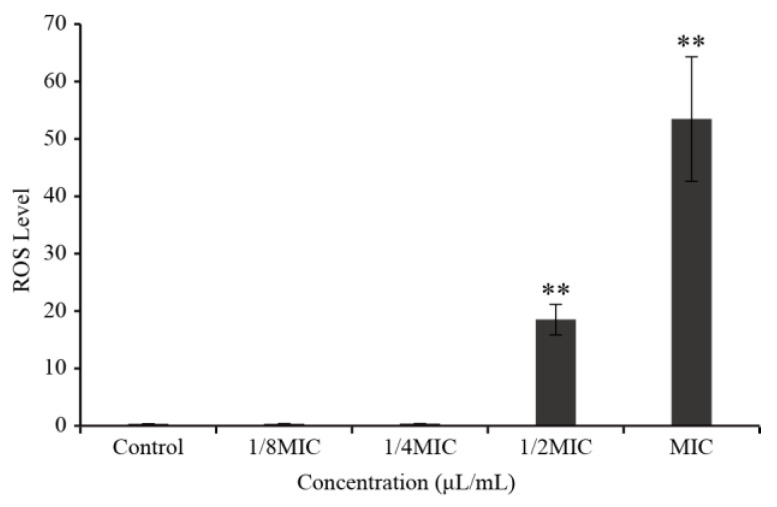
Intracellular ROS level in *C. sakazakii* treated with LC-EO (** *p* < 0.01).

ROS levels in *C. sakazakii* treated with LC-EO are shown in Figure 2. The level of ROS was lower in Control cells and cells treated with low concentrations of LC-EO (1/8MIC and 1/4MIC). However, ROS generation values increased significantly (*p* < 0.01) with increasing LC-EO concentration—compared with the Control, it increased by 1234 times (1/2MIC) and 3567 times (MIC), respectively.

### 3.4. CLSM-Based Observations

**Figure 3:**
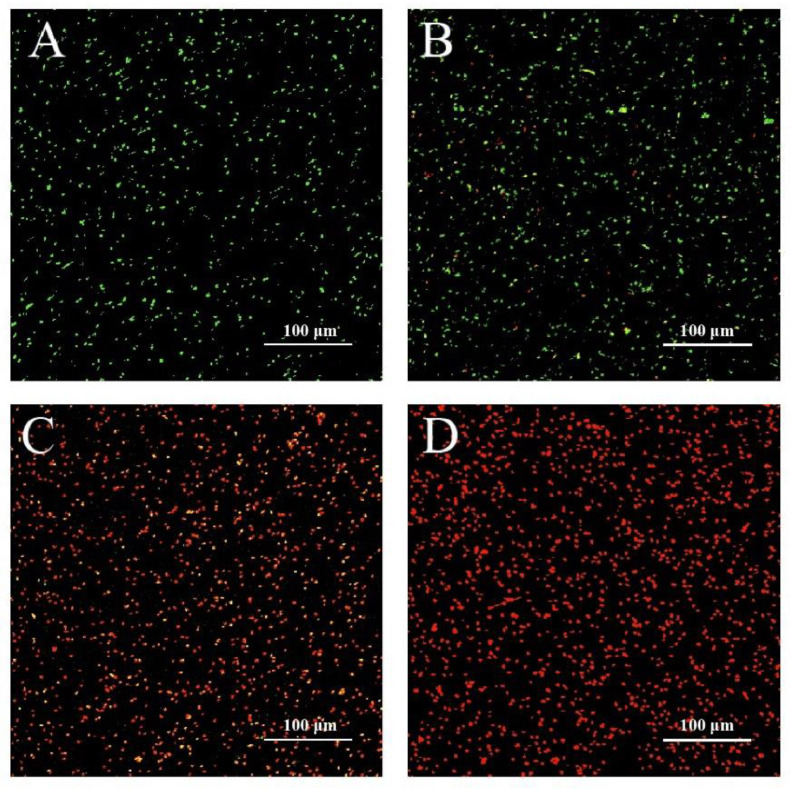
Integrity of the cell membrane of *C. sakazakii* treated with LC-EO observed by CLSM: (**A**) 0 (Control); (**B**) 1/4MIC; (**C**) 1/2MIC; (**D**)MIC.

Cell membrane permeability was reflected by the CLSM images (Figure 3). Green fluorescence was emitted from bacterial cells without LC-EO treatment (Control). A small amount of red fluorescence was observed from the treatment with 1/4MIC of LC-EO. With increasing LC-EO concentration, the intensity of red fluorescence increased gradually. After incubation with LC-EO at MIC for 30 min, red fluorescence was emitted by almost all cells.

### 3.5. Membrane Potential

**Figure 4:**
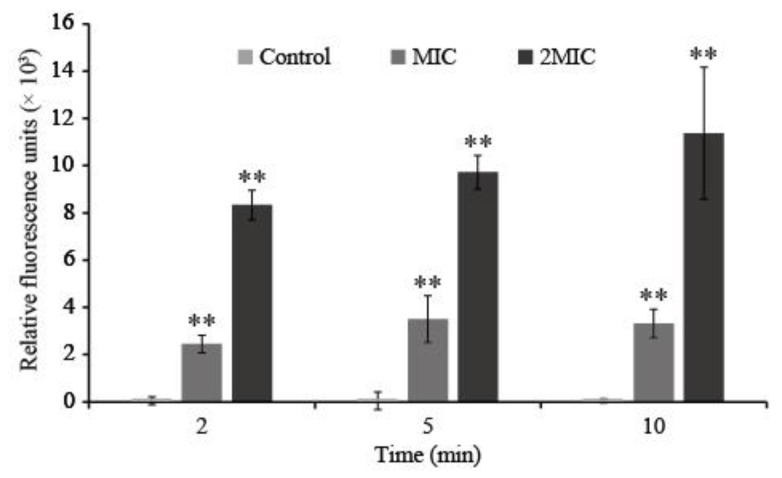
Cell membrane potential of *C. sakazakii* treated with LC-EO (** *p* < 0.01).

After being treated with LC-EO for 2 min, the membrane potential of *C. sakazakii* depolarized in an LC-EO-concentration-dependent manner (Figure 4). The depolarization became more obvious with the increase in treatment time from 2 to 10 min.

### 3.6. Intracellular ATP

The calibration curve between the relative fluorescence intensity and the concentration of ATP in standard solutions showed good linearity (y = 321319x + 1537; R^2^ = 0.999; data not shown).

**Figure 5:**
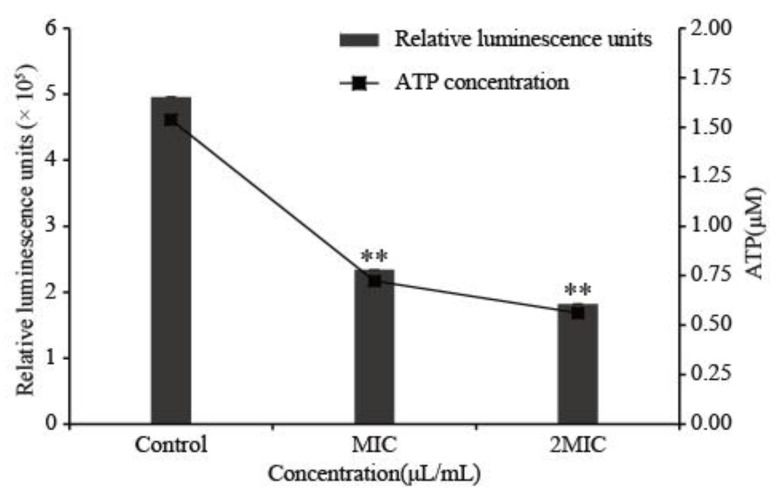
Intracellular ATP content of *C. sakazakii* treated with LC-EO (** *p* < 0.01).

A highly significant decrease in intracellular ATP in *C. sakazakii* (*p* < 0.01) was observed with LC-EO treatment in an LC-EO-concentration-dependent manner (Figure 5). The intracellular ATP concentration was 1.54 ± 0.03 μmol/L in the Control group, and 0.72 ± 0.02 μmol/L (MIC) and 0.56 ± 0.01 μmol/L (2MIC) in the treatment groups.

### 3.7. FESEM-Based Observations

**Figure 6:**
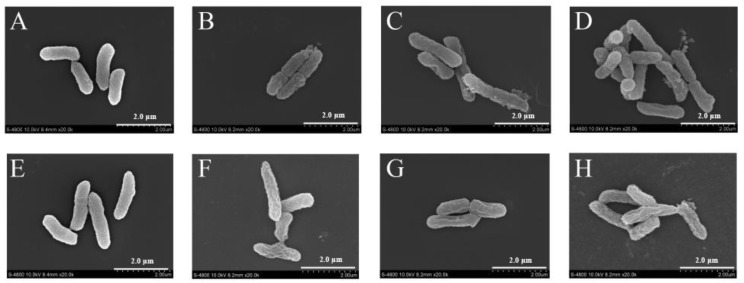
FESEM images of *C. sakazakii* with LC-EO treatment at 0MIC for 0.5 h (**A**), 1/4MIC for 0.5 h (**B**), 1/2MIC for 0.5 h (**C**), MIC for 0.5 h (**D**), 0MIC for 1 h (**E**), 1/4MIC for 1 h (**F**), 1/2MIC for 1 h (**G**), and MIC for 1 h (**H**).

Figure 6 displays FESEM images of *C. sakazakii* ATCC 29004 treated with LC-EO. Untreated cells were plump and rod-shaped with a smooth surface (Figure 6A,E). After treatment with LC-EO at 1/4MIC for 0.5 h, the cells shrank (Figure 6B). As the concentration of LC-EO increased from 1/4MIC to 1/2MIC, the degree of shrinkage increased (Figure 6C). After treatment with LC-EO at MIC for 0.5 h, the cells showed extensive surface collapse (Figure 6D). The morphological changes were more notable as the treatment time increased from 0.5 to 1 h (Figure 6F–H).

### 3.8. Antibacterial Activity of LC-EO toward C. sakazakii in TSB and PBS

**Figure 7:**
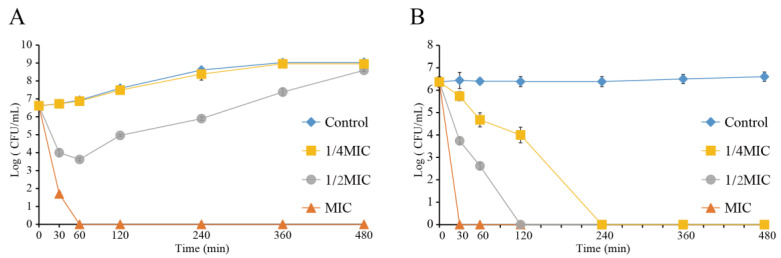
Effect of LC-EO on *C. sakazakii* populations in TSB (**A**) and PBS (**B**).

Figure 7A shows the antibacterial activity of LC-EO toward *C. sakazakii* in TSB. The initial cell concentration was 6.6 log CFU/mL. The bacterial concentration in the Control group and the group treated with 1/4MIC grew slowly over 8 h to 9.0 log CFU/mL. Upon treatment with LC-EO at 1/2MIC, the population of *C. sakazakii* decreased briefly, then began to rise again after 60 min, and thereafter the bacterial concentration increased. Upon treatment with LC-EO at MIC, bacterial counts were below the detection limit (1 CFU/mL) by 60 min.

Figure 7B shows the antibacterial activity of LC-EO toward *C. sakazakii* in PBS. The initial concentration of cells was 6.6 log CFU/mL, and the bacterial concentration in the Control group remained at this level. With the increase in the concentration of LC-EO and extension of the treatment time, the cell population decreased. The population of *C. sakazakii* dropped below the detection limit after treatment with LC-EO at 1/4MIC, 1/2MIC, and MIC for 240, 120, and 30 min, respectively.

### 3.9. Anti-C. sakazakii Activity upon Combined Treatment with LC-EO and Mild Heat in RIF

**Figure 8:**
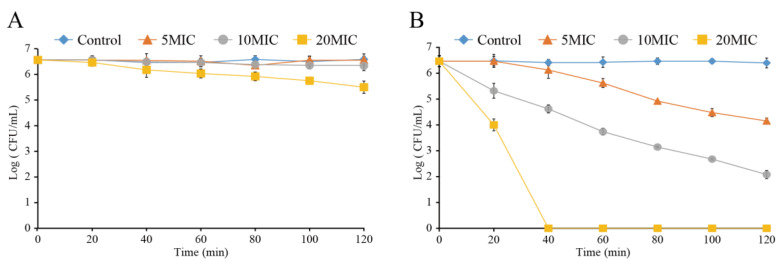
Effect of LC-EO on *C. sakazakii* populations in RIF at 25 °C (**A**) and 50 °C (**B**).

The inhibitory effect of LC-EO on *C. sakazakii* in RIF at 25 °C (Figure 8A) and 50 °C (Figure 8B) was investigated. The initial concentration of *C. sakazakii* in RIF at 25 °C was about 6.6 log CFU/mL (Figure 8A), and the number of viable cells in the Control remained at this level for 120 min. After treatment with LC-EO at 5MIC and 10MIC for 120 min, the bacterial counts hardly decreased, while the concentration of *C. sakazakii* decreased by 1.1 log CFU/mL after 120 min treatment with 20MIC LC-EO.

Figure 8B shows that there was no marked change in the number of bacteria in the Control group within 120 min at 50 °C. The total *C. sakazakii* population decreased by 2.6 and 4.6 log CFU/mL after being exposed to LC-EO at 5MIC and 10MIC for 120 min, respectively. After treatment with LC-EO at 20MIC for 40 min, the total population of *C. sakazakii* decreased below the detection limit (1 CFU/mL).

### 3.10. Inactivation Effect of LC-EO on C. sakazakii in Biofilms

**Figure 9:**
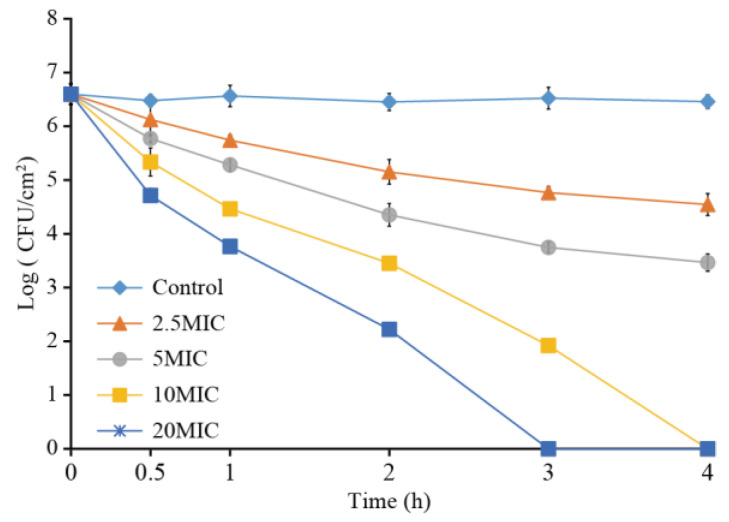
The number of viable *C. sakazakii* cells in biofilms on stainless steel after LC-EO treatment.

Figure 9 shows the effectiveness of LC-EO in the inactivation of viable bacteria in *C. sakazakii* biofilms. The number of bacteria in the untreated biofilm remained constant, at approximately 6.5 log CFU/cm^2^, for 120 min. The biofilm population of *C. sakazakii* was reduced to the detection limit (1 CFU/cm^2^) at 4 h and 3 h upon treatment with 10MIC and 20MIC of LC-EO, respectively. LC-EO concentrations of 2.5MIC and 5MIC did not completely inactivate the biofilm within 4 h, but the numbers of bacteria in the biofilm respectively reduced by 1.9 and 3.0 log CFU/cm^2^.

### 3.11. ATR-FTIR-Based Analysis of the Antibiofilm Activity of LC-EO

**Figure 10:**
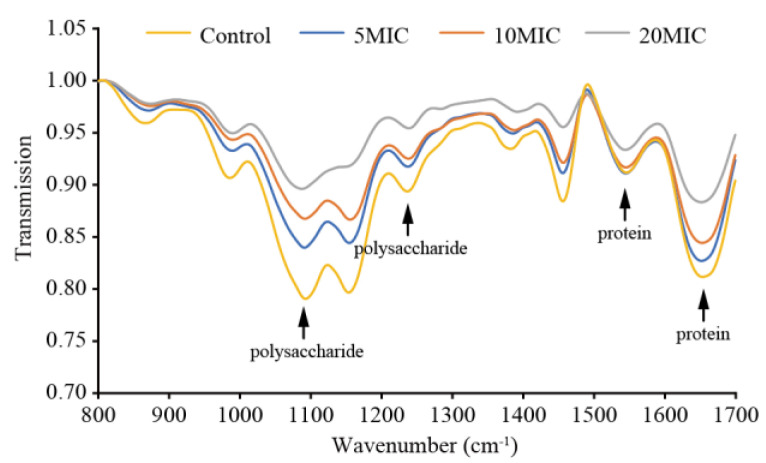
ATR-FTIR spectra of *C. sakazakii* biofilms on stainless steel.

The effects of LC-EO on biofilm components were observed via ATR-FTIR (Figure 10). There were absorption peaks near 854, 976, 1084, 1146, 1243, 1371, 1450, 1540, and 1650 cm^−1^, and the intensity of the peaks decreased as the concentration of LC-EO increased. The characteristic peaks near 1540 and 1650 cm^−1^ represent the amide I and amide II bands from protein, respectively, while the peaks near 1084 and 1243 cm^−1^ are characteristic of polysaccharides. The results demonstrate that the content of protein and polysaccharide in the biofilm decreased significantly after treatment with LC-EO, and as the treatment concentration increased, the level of protein and polysaccharide in the biofilm declined.

### 3.12. CLSM-Based Analysis of the Antibiofilm Activity of LC-EO

**Figure 11:**
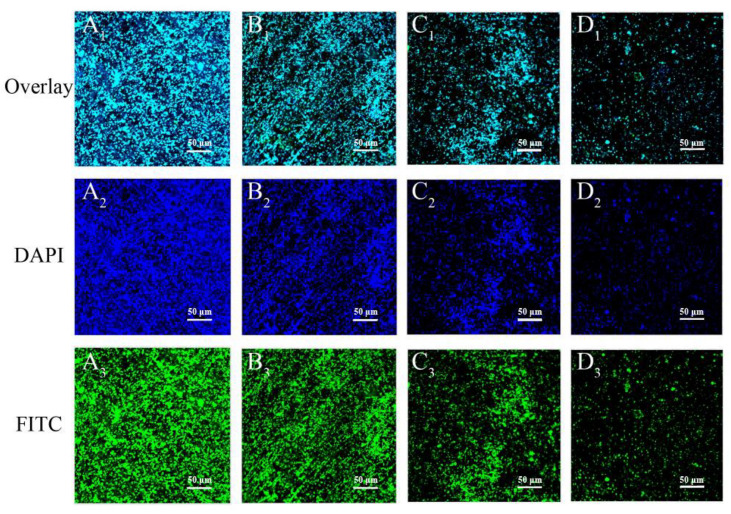
CLSM images at 40× magnification of *C. sakazakii* biofilms treated with LC-EO at 0 MIC (**A_1_**–**A_3_**), 5 MIC (**B_1_**–**B_3_**), 10 MIC (**C_1_**–**C_3_**), 20 MIC (**D_1_**–**D_3_**).

CLSM was employed to visualize the anti-*C. sakazakii* biofilm effects of LC-EO (Figure 11). Untreated biofilms (Figure 11A_1_–C_1_) showed dense green and blue fluorescence, indicating intensely distributed biofilm architectures. With the increase in LC-EO concentration from 0 to 20 MIC, the green and blue fluorescence decreased remarkably (Figure 11D_1_,A_2_–D_2_,A_3_–D_3_), which indicates that polysaccharides were removed significantly, and the bacterial numbers in the biofilm had reduced.

## 4. Discussion

In this work, LC-EO was shown to exhibit inhibitory efficacy toward eight *C. sakazakii* strains, with MIC values ranging from 1.5 to 4.0 μL/mL (approximately 1.32–3.53 mg/mL, Table 1). Previous research has determined the antibacterial activities of other natural substances toward *C. sakazakii*. Park et al. [29] determined that the MIC of *Lavandula* extract toward *C. sakazakii* was 25 μL/mL; Chang et al. [30] showed *Chrysanthemum buds* crude extract MIC values of 10 to 20 mg/mL for eight *C. sakazakii* strains; meanwhile, the MIC values of ferulic acid toward different *C. sakazakii* strains in the range of 2.5–5 mg/mL [31]. Therefore, in comparison with the above-cited literature data, LC-EO had the stronger inhibitory activity towards *C. sakazakii*. Liu and Yang [32] used gas chromatography to separate the main components of LC-EO, and found that they were citral (69.8%), limonene (12.7%), and linalool (1.4%), each of which has antibacterial activity. It was reported that the MIC of citral toward *C. sakazakii* CICC 21544 was 0.8 mg/mL [33], and the MIC values of limonene and linalool toward *C. sakazakii* ATCC 29544 were 3.0 and 2.0 mg/mL, respectively [34]. Therefore, citral plays a major antibacterial role in LC-EO. Although the antibacterial effect of LC-EO is weaker than that of pure citral, the use of LC-EO is more economical because LC-EO does not require complex purification. Thus, LC-EO is a low-cost antibacterial substance, which has potential for application in the food industry.

As shown in this study, LC-EO increased the intracellular ROS production in *C. sakazakii*, and the increase was more significant at high concentrations of LC-EO (Figure 2). As reported by Sun et al. [35], the treatment of *Staphylococcus aureus* cells with 2,4-dichlorobenzyl derivative 7d for 2 h induced the generation of intracellular ROS. ROS production proves that this substance possesses the potential for perturbing bacterial metabolism. Shivaprasad et al. [36] found that treatment of *E. coli* with vitamin C, a powerful antibacterial agent, led to increased ROS production, which accelerated the destabilization and disruption of the bacterial membrane, causing the release of intracellular biomolecules. Dai et al. [18] showed that three key enzyme activities of the tricarboxylic acid (TCA) cycle of *E. coli* were inhibited by LC-EO, which may stimulate the production of ROS. Some apoptotic signals, such as the change in Ca^2+^ concentration, are followed by ROS generation, which can induce further apoptosis [37]. Excessive ROS may disrupt the membrane integrity and cause impairment in membrane permeability [38].

In this study, the cell membrane integrity of *C. sakazakii* was assessed after treatment with LC-EO. The number of cells with damaged membranes increased along with the increased LC-EO concentration from 1/4MIC to 1MIC (Figure 3). Similarly, Tian et al. [39] reported that after *C. sakazakii* was incubated with thymol at 2MIC, there was almost no green fluorescence emitted by SYTO 9, indicating that the cell membrane integrity was significantly compromised. Subramaniyan et al. [26] used the fluorescence probe acridine orange/PI to observe *S. aureus* treated with silver nanoparticles; the number of cells emitting red fluorescence significantly increased after treatment with the nanoparticles at MIC, illustrating that the cell membrane was damaged. Combined with the results for the analysis of ROS levels, we suggest that upon treatment of *C. sakazakii* with LC-EO, lipid oxidation of the cell membrane was accelerated by excessive ROS, leading to damage to membrane integrity.

The potential difference between the two sides of the cell membrane at rest is called membrane potential. It is an important sign of the vital state of the bacterial cells. Membrane potential is essential for the absorption of antimicrobial agents and bactericidal action [40]. Our results show that LC-EO induced the depolarization of the *C. sakazakii* cell membrane (Figure 4). Similar results have been detected in recent studies on lipoic acid treatment of *C. sakazakii* [41] and *Amaranthus tricolor*-derived crude extract treatment of *S. aureus* [42]. However, Shi et al. [31] reported that ferulic acid hyperpolarized the *C. sakazakii* cell membrane. Some studies found that the hyperpolarization of the membrane occurs as a result of the alteration of pH and the movement of K^+^ across the membrane, and depolarization is mainly related to the opening of Na^+^ channels [43,44].

ATP concentration is a crucial parameter that reflects the vital status of cells [45]. In this assay, LC-EO significantly decreased the intracellular ATP concentration of *C. sakazakii* (Figure 5). Similar findings were reported by Shi et al. [41]; lipoic acid at MIC and 2MIC significantly decreased the ATP concentration in *C. sakazakii*. Guo et al. [7] showed that ATP was significantly decreased in *C. sakazakii* upon treatment with coenzyme Q0, in a concentration-dependent manner. Consistent with the above research, Shi et al. [25] suggested that after treatment with natural products, increased cell membrane permeability led to the release of ATP. Additionally, some natural products were found to alter bacterial metabolism, such as dysregulating the TCA cycle, to inhibit ATP formation [46]. Moreover, the hydrolysis of ATP was promoted by the leakage of inorganic phosphate through a damaged cell membrane and the subsequent attempt to restore the electrochemical gradient was driven by the consumption of ATP [47].

FESEM was used to evaluate the cell membrane morphology. After exposure to LC-EO, *C. sakazakii* cells were deformed (Figure 6). Similar studies have also confirmed the activity of natural substances on the morphology of foodborne pathogens. Shi et al. [23] reported that citral at 2MIC caused severe morphological alterations in *C. sakazakii*. Zou et al. [48] indicated that the shrinkage and deformation of *E. coli* and *S. aureus* cells caused by black pepper chloroform extract may be related to the leakage of low-molar-mass metabolites after a change in membrane permeability. According to above studies, *C. sakazakii* treated with LC-EO showed serious damage to cellular integrity, followed by the loss of intracellular material, leading to morphological change [12].

Survival counts of *C. sakazakii* in TSB and PBS after treatment with LC-EO decreased with the extension of treatment time and increasing concentration of LC-EO (Figure 7). The killing effect of LC-EO on other bacteria also shows this trend. Time dependence and concentration dependence were characteristic of the reduction in drug-resistant *Acinetobacter baumannii*, as determined by measuring OD_600 nm_ at intervals [49]. Thielmann et al. [50] reported that on the surface of food packaging films and across the gas phase, LC-EO achieved bactericidal effects against *E. coli* in a time-dependent manner. In this study, the effect of LC-EO treatment was greater in PBS than in TSB (Figure 7). Similarly, the bacteriolysin lysostaphin and endolysin PlyPH were more effective at killing bacteria in PBS than in TSB [51]. Guo et al. [45] also found that the reduction in *Listeria monocytogenes* survival was quicker in normal saline than in Luria–Bertani broth when treated with olive oil polyphenol extract. Therefore, LC-EO has different antibacterial effects in nutrient media and non-nutrient media. In practical application, the dosage needs to be evaluated according to the medium.

Mild heating (at 50 °C) combined with LC-EO treatment was explored as an alternative method of reconstituting PIF; the recommendation of the Food and Agriculture Organization/the World Health Organization to reconstitute at >70 °C means that the feed is not suitable for immediate consumption [2]. Mild heating combined with LC-EO greatly enhanced the inhibitory activity on *C. sakazakii* in RIF (Figure 8). Similarly, the results of Gabriel and Pineda [52] showed that vanillin and licorice root extract effectively reduced the number of *E. coli* O157:H7 cells in coconut juice at 55 °C. Shi et al. [53] found that within a certain range, the bactericidal effect of thymoquinone in RIF was enhanced when the heating temperature was increased. According to Pedrosa et al. [54], treatment under such temperatures can cause DNA damage, loss of metabolic activity, and efflux activity in bacteria. Consequently, the use of combined LC-EO and mild heating possibly improved the effect of each, and this method has the potential to become an efficient means of food sterilization.

Bacterial biofilms, including of *C. sakazakii*, are difficult to control because the biofilm provides protection for the bacteria in the film [7]. Therefore, in order to eradicate biofilms in the food industry, considerable economic resources have been spent in the development of strategies [55]. In this study, LC-EO significantly reduced the number of viable bacteria in biofilms formed by *C. sakazakii* (Figure 9). The concentration of LC-EO needed was greater than the MIC for planktonic bacteria, as in other studies. According to Wang et al. [56], neither *S. aureus* nor methicillin-resistant *S. aureus* biofilm levels were significantly reduced by treatment with *Ginkgo biloba* L. exocarp extract at MIC and 2MIC. Guo et al. [7] found that treatment with 4 mg/mL (40MIC) of coenzyme Q0 for 120 min decreased the concentration of viable *C. sakazakii* in biofilms by 4.94 log CFU/cm^2^. To increase the antibiofilm effect of LC-EO, LC-EO combined with enzymes or acid–anionic tensioactive cleaning will be further explored.

A complete biofilm matrix, including proteins, phosphorus-containing carbohydrates, extracellular polysaccharides, and nucleic acids, plays a crucial role in the protection of pathogenic cells in the biofilms [27]. ATR-FTIR analysis showed that proteins and extracellular polysaccharides were significantly decreased in *C. sakazakii* biofilms after treatment with LC-EO, in a concentration-dependent manner (Figure 10). Similarly, Huang et al. [57] showed that at 25 °C, after light-emitting diode (LED) irradiation for 2 h, the protein and extracellular polysaccharide levels in biofilms of *C. sakazakii* were decreased. Vishwakarma and Sirisha [58] reported that a ninefold reduction in extracellular polysaccharide was observed when *Salmonella enterica* biofilm was treated with 0.44 mg/mL of *Chlamydomonas reinhardtii*-sulfated polysaccharides (Cr-SPs), and a threefold reduction in the biofilm was observed upon treatment with 2 mg/mL Cr-SPs.

Hoechst 33258 stains bacterial cells blue, and Con-A FITC stains exopolysaccharides green [28]. In the present study, as the concentration of LC-EO increased, the blue and green fluorescence from *C. sakazakii* decreased, indicating that the number of bacterial cells and the level of exopolysaccharide declined (Figure 11). Similarly, using CLSM images, Liang et al. [59] showed that after treatment with Ag_3_PW_12_O_40_ nanoparticles, a *S. aureus* biofilm was degraded into fragments, and the content of extracellular polysaccharide was significantly decreased. Yang et al. [28] used CLSM to observe the fluorescence of Hoechst 33258 and Con-A FITC in *Pseudomonas aeruginosa* biofilm, which was significantly decreased after LED irradiation, indicating that LED irradiation can cause a decrease in extracellular matrix components and viable bacteria. LC-EO can not only inactivate *C. sakazakii* cells in a biofilm, but also decrease extracellular substances in the biofilm.

## 5. Conclusions

LC-EO exhibits good antibacterial activity and biofilm scavenging activity toward *C. sakazakii*. LC-EO has the following mechanisms: prolonging the delay period of bacterial growth; increasing the ROS level; decreasing membrane integrity; and increasing the permeability of the cell membrane, causing cell morphological changes. LC-EO was able to kill both reference strains and isolates of *C. sakazakii* in planktonic form, using low concentrations of essential oil. LC-EO combined with mild heating (50 °C) inactivated *C. sakazakii* in RIF. LC-EO can reduce the number of *C. sakazakii* biofilm cells and the extracellular matrix on stainless steel surfaces. Therefore, LC-EO has the potential to act as a natural antibacterial agent in the food industry to control contamination by *C. sakazakii*. Some issues on slowing down the evaporation of LC-EO and the influence of LC-EO on the sensory properties of the food products need to be resolved in depth before its application.

## Figures and Tables

**Table 1:** MIC and MBC of LC-EO toward eight strains of *C. sakazakii*.

Strain	Origin	MIC (μL/mL)	MBC (μL/mL)
ATCC 29004	Infant formula	3.0	3.0
ATCC 29544	Children’s throat	3.0	3.0
ATCC 12868	Infant formula	3.0	4.0
ATCC BAA-894	Human clinical specimens	4.0	4.0
18-8(2)	Infant formula	3.0	3.0
14-15(1)	Infant formula	1.5	3.0
18-7(2)	Rice flour	4.0	6.0
11-8(2)	Rice flour	4.0	6.0

## Data Availability

The data presented in this study are available on request from the corresponding author.
